# Associations between self-esteem and smoking and excessive alcohol consumption in the UK: A cross-sectional study using the BBC UK Lab database

**DOI:** 10.1016/j.abrep.2019.100229

**Published:** 2019-10-19

**Authors:** Dorothy Szinay, Ildiko Tombor, Claire Garnett, Neil Boyt, Robert West

**Affiliations:** a School of Health Sciences, University of East Anglia, Norwich Research Park, ECB Building, NR4 7TJ, United Kingdom; b Department of Behavioural Science and Health, University College London, 1-19 Torrington Place, WC1E 6BT, United Kingdom

**Keywords:** Self-esteem, Smoking, Alcohol consumption

## Abstract

**Introduction:**

There is mixed evidence regarding the associations between self-esteem and smoking and excessive alcohol consumption. This study aimed to investigate whether self-esteem is associated with smoking status and alcohol consumption in a large sample of adults in the United Kingdom after adjusting for age, sex, socio-economic status and depressed mood.

**Methods:**

**Design:** Cross-sectional correlational study conducted under the aegis of the British Broadcasting Corporation (BBC) between 2009 and 2013.

**Participants:** 187,398 respondents (67.9% female) with a mean age of 32.82 years (SD = 12.41) providing complete data.

**Setting:** UK.

**Measures:**

Online self-report questionnaire. The outcomes were smoking status (current smoker, ever smoker) and alcohol consumption (current drinker, excessive drinker); the input variable was self-esteem measured using a Single Item Self-Esteem Scale; covariates were age, sex, SES, and depressed mood measured using a single item question.

**Results:**

The odds of being an ever smoker and a current smoker were greater in people with lower self-esteem (AdjOR 0.97; 95% CI 0.95–0.99, Cohen’s d = −0.02; and AdjOR 0.96; 95% CI 0.94–0.99, Cohen’s d = −0.02 respectively). The odds of being a current drinker were lower in people with lower self-esteem (AdjOR 1.20, 95% CI 1.17–1.24, Cohen’s d = 0.10) while being an excessive drinker was associated with lower self-esteem (β = −0.13, p < 0.001, F(5, 187392) = 997.14, p < 0.001, Cohen’s d = 0.3).

**Conclusions:**

Lower self-esteem appears to be positively associated with ever- and current smoking and excessive alcohol consumption and negatively associated with current alcohol consumption.

## Highlights

• Data were used from a large, cross-sectional study conducted in the UK.
• Associations between self-esteem and smoking and alcohol consumption were assessed.
• Analyses were adjusted for age, gender, socioeconomic status, and depressed mood.
• Lower self-esteem appears to be associated with smoking status.
• Lower self-esteem was associated with excessive alcohol consumption.

## 1 Introduction

Smoking and excessive alcohol consumption are major public health concerns and are prevalent in the UK. Globally, around one in seven adults are daily smokers and one in five adults consumes alcohol excessively at least once a month (Peacock et al., 2018). In 2017, 16.8% of the adult population in the UK smoked daily regularly (Office for National Statistics, 2018b) and the prevalence of excessive alcohol consumption, measured as alcohol consumption exceeding 12 (for men)/9 (for women) units of alcohol on their heaviest day in the previous week, was 15% (Office for National Statistics, 2018a).

The combination of smoking and alcohol consumption have a greater impact on the disease burden than either behaviour alone (Peacock et al., 2018) and beyond health consequences, bring significant social and economic losses to public health, such as ill-health related costs. Furthermore, in 2017, excessive alcohol consumption was more than twice as common among smokers (17%) than in never smokers (6%) or non-smokers (7%) in the UK (Office for National Statistics, 2018a). Hence it is not surprising that there is a need to identify factors that predict smoking and excessive alcohol consumption to help implement further efforts for smoking cessation and alcohol reduction.

Psychological factors, such as stress and depression, have been identified for behaviour change interventions for smoking cessation and to reduce alcohol consumption (Ockene et al., 2000). Self-esteem is another potentially important psychological factor in targeting addictive behaviours (Alavi, 2011), though it has not been studied extensively in terms of its associations with smoking and alcohol consumption. Global self-esteem is defined as an interpretation of one’s self-worth (Rosenberg, 1965), and the beliefs and perceptions about oneself in a favourable or unfavourable way (Baumeister, Campbell, Krueger, & Vohs, 2003). It has been suggested as an important motivational factor to drive behaviour (West & Brown, 2013), as people with high self-esteem are likely to feel good about themselves and engage in behaviours that protect or improve their health and wellbeing (Du et al., 2017, Wellman et al., 2016), whereas low self-esteem has been shown to be positively associated with substance use (Saari et al., 2015, Wellman et al., 2016).

However, the direction and magnitude of associations between measures of self-esteem and smoking and excessive alcohol consumption are not consistent in the literature. Some studies have found that self-esteem is not associated with smoking (Baumeister et al., 2003); a few survey studies have shown that low self-esteem in younger age might predict smoking later in life (Andrews and Duncan, 1997, Jackson et al., 1997, Pederson et al., 1998), and that low self-esteem girls are more likely to try smoking, than boys with low self-esteem (Abernathy et al., 1995, Lewis et al., 2001). A longitudinal study in Finland conducted on adolescents also found that self-esteem was not associated with smoking (Saari et al., 2015). However, several large studies included in the review of Baumeister and colleagues showed no relationship between self-esteem and smoking in adolescents and young adults (Glendinning, 1998, McGee and Williams, 2000). Other systematic reviews have found a positive association between low self-esteem and smoking in adolescents (Chapman and Wu, 2013, Wellman et al., 2016). However, a systematic review has pointed out a different role self-esteem may play, that is people with high self-esteem are more likely to quit smoking compared with those with low self-esteem (Freijy & Kothe, 2013).

In terms of the association between self-esteem and excessive alcohol consumption, the narrative review conducted by Baumeister et al. (2003) included studies with highly rigorous methods that examined the casual impact of self-esteem on personal and social problems. The review reported self-esteem as being an inconsistent predictor of excessive alcohol consumption. The studies included in the review that found a link between self-esteem and excessive alcohol consumption pointed in different directions: some studies linked alcohol consumption to low self-esteem (Jackson et al., 1997, Li and Moore, 1998), whilst others to high self-esteem (Glendinning, 1998, Griffin et al., 2000). However, it was found that high self-esteem was associated with less excessive alcohol consumption among college students in the USA (Backer-Fulghum, Patock-Peckham, King, Roufa, & Hagen, 2012), while findings from a large cross-sectional study of adults in China showed that low self-esteem was related to excessive alcohol consumption (Zhai et al., 2015).

Previous research has also indicated that self-esteem may be associated with various sociodemographic characteristics, such as age (Trzesniewski, Donnellan, & Robins, 2003), gender (Blank et al., 2016, Hale et al., 2015, Veselska et al., 2009), and socioeconomic status (SES) (Twenge and Campbell, 2002, Twenge and Crocker, 2002). Moreover, studies conducted in the USA showed that people with mental health problems, such as depression and anxiety, are more likely to as have low self-esteem (Sowislo and Orth, 2013, Tucker et al., 2011) as well as smoke (Tucker et al., 2011, Williams and Ziedonis, 2004) and consume alcohol excessively (Grant, Hasin, Chou, Stinson, & Dawson, 2004).

Overall, the findings regarding the associations between self-esteem and smoking and excessive alcohol consumption is conflicting. The majority of studies have been conducted on a small scale and on specific populations, such as adolescents, students, mothers, or problem drinkers (Chapman and Wu, 2013, Hale et al., 2015, Saari et al., 2015, Veselska et al., 2009, Wellman et al., 2016, Zhai et al., 2015). To the authors’ knowledge the relationship between self-esteem and smoking and alcohol consumption has never been assessed with a large study among the general population of adults in the UK. This paper aimed to investigate these associations in a large sample of adults in the UK. The study addressed the following research questions: What are the associations between self-esteem and (1) smoking status and (2) alcohol consumption among adults in the UK, whilst adjusting for sociodemographic characteristics and depressed mood.

## 2 Methods

This study was a secondary data analysis of an online cross-sectional study conducted by the British Broadcasting Corporation (BBC). The dataset was fully anonymised. The study protocol including the analysis plan was preregistered on Open Science Framework prior to data analysis (https://osf.io/q4phz/).

### 2.1 Participants

Participants were aged 16 and over and resided in the UK. Participants with missing data on the variables examined were excluded from the study.

### 2.2 Procedure

Participants were recruited by the BBC between 2009 and 2013. The BBC invited members of the public to take part in online surveys and experiments on their website (http://www.bbc.co.uk/labuk). The surveys and experiments were advertised on BBC media outlets, such as their website, radio and television shows (Rentfrow, Jokela, & Lamb, 2015). The studies, for example the “Big Personality Test” (Rentfrow et al., 2015), were designed by academic researchers and targeted different aspects of psychology, sociology and health (Morrissey, Kinderman, Pontin, Tai, & Schwannauer, 2016). Ethical approval was obtained by the researchers who conceived the studies (Lane et al., 2016). Every participant had a unique identifier which allowed them to participate only once. All participants gave their consent prior to participation (Morrissey et al., 2016) and online feedback was provided on their results (Rentfrow et al., 2015).

### 2.3 Measures

#### 2.3.1 Predictor variable

##### 2.3.1.1 Self-esteem

Self-esteem was assessed by a Single-Item Self-esteem Scale (SISE): ‘I see myself as someone with high self-esteem’ (from 1: disagree strongly to 5: agree strongly), and for ease of interpretation was dichotomised into ‘high’ self-esteem (1) (‘agree strongly’ and ‘agree a little’) and ‘low’ self-esteem (0) (‘disagree strongly’, ‘disagree a little’ and ‘neither agree nor disagree’). It has been shown that SISE has similar convergent and predictive validity to the widely used Rosenberg Self-esteem Scale (Robins, Hendin, & Trzesniewski, 2001), although internal consistency reliability cannot be computed for a single-item scale.

#### 2.3.2 Outcome variables

##### 2.3.2.1 Smoking status

Ever smoking status was assessed by asking participants ‘Have you ever smoked cigarettes daily, that is, at least one cigarette every day for 30 days?’. Those who answered ‘yes’ were defined as ever smokers (1), whilst those who answered ‘no’ as never smokers (0). Current smoking status was assessed by ‘During the past 30 days, on average how many cigarettes did you smoke per day?’ with seven possible answers: ‘none’, ‘less than 1 cigarette per day’, ‘1 cigarette per day’, ‘2–5 cigarettes per day’, ‘6–10 cigarettes per day’, ‘11- 20 cigarettes per day’, ‘more than 20 cigarettes per day’). Those who answered ‘none’ were defined as ‘non-smoker’ (0); others were defined as ‘current smoker’ (1). Ever smoking status and current smoking status were chosen as they are the most common smoking variables used in epidemiologic studies related to smoking status (Leffondré et al., 2002, Pruchno et al., 2012).

##### 2.3.2.2 Drinking status

Current alcohol consumption was assessed by ‘During the past 30 days, on how many days did you have at least one drink of alcohol?’ (‘0 day’, ‘1 or 2 days’, ‘3–5 days’, ‘6–9 days’, ‘10–19 days’, ‘20–29 days’, ‘all 30 days’). A binary variable was derived and those who answered ‘0 day’ were defined as ‘non-drinker (0); others were defined as ‘current drinker’ (1).

Excessive alcohol consumption was defined as exceeding 12 (male) or 9 (female) units of alcohol on their heaviest drinking day (ONS, 2018b), that is roughly equal to 5 standard drinks commonly consumed in the UK, for example 5 pints of beer at 5.2% alcohol by volume. Excessive alcohol consumption was assessed by ‘During the past 30 days, on how many days did you have 5 or more drinks of alcohol in a row, that is, within a couple of hours?’ (‘0 day’, ‘1 day’, ‘2 days’, ‘3–5 days’, ‘6–9 days’, ‘10–19 days’, ‘20 or more days’). This variable was transformed into a continuous variable and, where necessary, the average of the range was calculated (0, 1, 2, 4, 8, 15, 20).

#### 2.3.3 Covariates

##### 2.3.3.1 Demographic characteristics

Age, gender and SES were assessed. SES was based on social grade and dichotomised into high SES (1) (non-routine and manual social grade) and low SES (0) (routine and manual social grade and unemployed/on state benefit).

##### 2.3.3.2 Depressed mood

Depressed mood was assessed by a single-item measure ‘I see myself as someone who is depressed, blue’, with answers ranging from 1: disagree strongly to 5: agree strongly and was dichotomised in ‘high’ (0) (‘agree strongly’, ‘agree a little’) and ‘low’ (1) (‘disagree strongly’, ‘disagree a little’, ‘neither agree nor disagree’). It has been shown that a single item measure of mood is a reasonable substitute for a longer assessment tool (McKenzie and Marks, 1999, Skoogh et al., 2010).

## 3 Analysis

The statistical analyses were conducted in IBM SPSS Statistics version 23.0. Frequency, mean and standard deviation were calculated for categorical and continuous variables respectively, to describe the sample.

### 3.1 Association between self-esteem and smoking

Binomial unadjusted logistic regression was conducted separately for both ever smoking status and current smoking status outcome variables with self-esteem as a predictor. The models were followed by adjusted logistic regression with age, gender, SES, and depressed mood as covariates.

### 3.2 Association between self-esteem and excessive drinking

Current drinking status was assessed by binomial unadjusted logistic regression with self-esteem as a predictor. An adjusted logistic regression model was fitted controlling for age, gender, SES, and depressed mood on drinking behaviour. Finally, linear regression was conducted with self-esteem as predictor and excessive drinking as an outcome variable, adjusted for age, gender, SES and depressed mood. The assumption of linear regression needed a non-linear transformation of the dependent variable and cube root transformation of the dependent variable was conducted.

## 4 Results

### 4.1 Participant characteristics

187,379 participants from the BBC dataset (total of 588,014 people) met the inclusion criteria for this study. Participants’ baseline characteristics are reported in Table 1. The mean age was 32.8 years (SD = 12.4), 67.9% (N = 127,164) were female and 61.6% (N = 115,481) were of high SES. Of all participants, 27.5% (N = 51,502) reported high depressed mood and 53.9% (N = 101,074) low self-esteem. One third of the sample were ever smokers (32.1%, N = 60,085) and 20.9% (N = 39,209) were current smokers. Only 18% (N = 33,644) of the participants were non-drinkers. The mean number of days for having more than five drinks in the past 30 days was 2.0 (SD = 3.6) and the median was 4 (IQR = 1–15).

**Table 1:** Characteristics of the sample (n = 187,398).

Age, mean (SD)	32.8 (12.41)
% female, (n)	67.9 (127,164)
% low SES (n)	38.4 (71,917)
% low depressed mood, (n)	72.5 (135,896)
% low self-esteem (n)	53.9 (101,074)
% ever smoker, (n)	32.1 (60,085)
% current smoker, (n)	20.9 (39,209)
% current drinker, (n)	82.0(153,754)
Number of days having 5 or more drinks in a row, mean (SD)	2.0 (3.6)

### 4.2 Associations between self-esteem and smoking

The unadjusted logistic regression model showed that having lower self-esteem was significantly associated with being an ever smoker (OR 0.92; 95% CI 0.91–0.94) and being a current smoker (OR 0.86; 95% CI 0.84–0.88). Lower self-esteem remained as a significant predictor in the adjusted logistic regression model after adjusting for gender, age, SES and depressed mood (AdjOR 0.97; 95% CI 0.95–0.99; and AdjOR 0.96; 95% CI 0.94–0.99) (see Table 2). In both cases the calculated effect size using Cohen’s d measure was very small d = −0.02.

**Table 2:** Association between self-esteem and smoking (n = 187,398).

	Ever smoker vs. never smoker			Current smoker vs. non-smoker		
Predictor	Adj. OR*	95% CI	p-value	Adj. OR *	95% CI	p-Value
Self-esteem (low = 1)	0.97	0.95; 0.99	0.005	0.96	0.94; 0.99	0.001
Age	1.03	1.03; 1.03	<0.001	0.99	0.99; 0.99	<0.001
Gender (male = 1)	0.85	0.83; 0.86	<0.001	0.77	0.75; 0.79	<0.001
SES (high = 1)	1.17	1.14; 1.19	<0.001	1.281	1.251–1.312	<0.001
Depressed mood (low = 1)	1.35	1.32; 1.38	<0.001	1.465	1.428–1.503	<0.001

* Adjusted for all study variables.

### 4.3 Associations between self-esteem and current drinking

The unadjusted logistic regression model showed that higher self-esteem was significantly associated with being a current drinker (OR 1.32; 95% CI 1.29–1.36) (see Table 3). After adjusting for gender, age, SES and depressed mood higher self-esteem remained a significant predictor (AdjOR 1.20, 95% CI 1.17–1.24) (see Table 3.). The effect size of the association was small d = 0.10.

**Table 3:** Association between self-esteem and current drinking (n = 187,398).

Predictor	Adj. OR*	95% CI	p-Value
Self-esteem (low = 1)	1.2	1.14; 1.24	<0.001
Age	1	1.00–1.00	<0.001
Gender (male = 1)	0.71	0.69; 0.73	<0.001
SES (high SES = 1)	0.64	0.62: 0.65	<0.001
Depressed mood (low = 1)	0.92	0.90; 0.95	<0.001

* Adjusted for all study variables.

### 4.4 Associations between self-esteem and excessive drinking

The results of the linear regression analysis are reported in Table 4, and showed that lower self-esteem (β = −0.15, P < 0.001) is associated with more days of excessive drinking (F(1, 187396) = 238.6, p < 0.001). In multiple regression adjusted for age, gender, SES and depressed mood (F(5, 187392) = 2201.59, p < 0.001) lower self-esteem (β = −0.13, p < 0.001) remained a significant predictor together with age, gender and depressed mood. A small effect size was observed d = 0.30.

**Table 4:** Association between self-esteem and excessive drinking (n = 187,398).

		B	95% CI	SE (B)	β	p
Model 1	Constant	2.05	2.03; 2.08	0.012		<0.001
	Self-esteem (low = 1)	−0.15	−0.18; −0.11	0.017	−0.020	<0.001

Model 2	Constant	2.55	2.49; 2.61	0.031		<0.001
	Self-esteem (low = 1)	−0.13	−0.17; −0.10	0.017	−0.018	<0.001
	Age	−0.02	−0.02; −0.02	0.001	−0.062	<0.001
	Gender (male = 1)	1.08	1.05; 1.12	0.018	0.141	<0.001
	SES (high = 1)	0.04	0.03; 0.04	0.018	−0.002	0.35
	Depressed mood (low = 1)	−0.04	−0.05; −0.03	0.019	−0.042	<0.001

Note. R^2^ = 0 for model 1; R^2^ = 0.026 model 2, ΔR^2^ = 0.026.

### 4.5 Exploratory sensitivity analysis

Tobacco and alcohol co-use is common (Daw, Nowotny, & Boardman, 2013) though the pre-registered analysis plan did not plan to adjust for one behaviour as a potential confounder when investigating the association between self-esteem and the other behaviour. Therefore, an additional exploratory sensitivity analysis was conducted to investigate the association between self-esteem and smoking, and self-esteem and excessive drinking, adjusting for the two co-use factors: excessive alcohol consumption and smoking, respectively, as well as the pre-specified covariates (age, gender, SES, depressed mood). The pattern of results was the same in this sensitivity analysis.

## 5 Discussion

There was a small difference in self-esteem between ever smokers and never smokers, and excessive drinkers and non-drinkers. People with lower self-esteem appeared more likely to be ever smokers and current smokers, and to consume alcohol excessively, while people with higher self-esteem appeared more likely to be current drinkers.

Previous research suggested that low self-esteem was associated with smoking in adolescents (Saari et al., 2015) and pregnant mothers (Chapman & Wu, 2013), and that high self-esteem would protect individuals against smoking (Wellman et al., 2016). This large study of the general adult population in the UK found similar results: lower self-esteem was associated with being an ever smoker and a current smoker. However, the effect size of these associations were incredibly small (Funder & Ozer, 2019). A potential explanation for these associations would be that people with more social support have higher self-esteem, than those who do not (Budd, Buschman, & Esch, 2009), and if that peer group smokes, then this might be associated with smoking uptake (Harakeh and Vollebergh, 2013, Stewart-Knox et al., 2005). Individuals with low self-esteem tend to be lonelier (Vanhalst, Luyckx, Scholte, Engels, & Goossens, 2013). A systematic review including studies on different populations with large, nationally representative samples, has found that loneliness is associated with smoking in both, adolescents and adult population (Dyal & Valente, 2015). Furthermore, young people are more vulnerable to substance use (Baumeister et al., 2003, Gerrard et al., 2000, Khajehdaluee et al., 2013) and by tending to minimise their susceptibility with cognitive strategies they might ignore possible harmful consequences of the risky behaviour. Therefore, despite the well-known health consequences of smoking, they may convince themselves that those consequences could not happen to them (Gerrard et al., 2000), and therefore, continue to smoke.

Conversely, in the case of current smoker status, a plausible explanation might lie in the effect of the anti-tobacco campaigns that have contributed to create a stigma regarding smoking. Evidence suggests that in recent decades smoking stigma has increased (Evans-Polce et al., 2015, Riley et al., 2017). During anti-smoking campaigns, public health practitioners often assume that smokers will internalise the smoking stigma created by these campaigns, eventually causing them to quit (Evans-Polce et al., 2015). However, one of the potential outcomes of the public stigma of smoking is that individuals might resist internalising smoking stigma and by becoming defensive it may decrease their self-esteem and self-efficacy even more and, most importantly, fail to quit smoking (Evans-Polce et al., 2015).

In terms of self-esteem as a predictor of excessive alcohol consumption, previous studies have reported mixed results. In a narrative review, self-esteem was found to be irrelevant to excessive alcohol consumption (Baumeister et al., 2003), while other studies found that low self-esteem was related to excessive alcohol consumption in the USA and China (Backer-Fulghum et al., 2012, Zhai et al., 2015). In line with the latter findings, the results of the current study showed that people with lower self-esteem are more likely to consume alcohol excessively. A possible reason behind the findings is that excessive alcohol consumption might be a coping mechanism of negative wellbeing represented by low self-esteem. According to the self-determination theory, self-esteem is secured when individuals are fully functioning, motivated and their needs are satisfied (Ryan & Deci, 2004). The theory also claims that when self-esteem is not secured, it becomes fragile and unstable, leading to unfavourable effects on wellbeing (Ryan & Deci, 2004) that might require pursuing activities, such as excessive alcohol consumption, which would attenuate the negative outcomes.

An important consideration in terms of how meaningful the findings of this study are is the effect size. The effect size of the association between self-esteem and ever smokers and current smokers was extremely small (d = 0.02); and the effect size of the associations between self-esteem and excessive drinking was small (d = 0.30). Although even very small effect sizes can be important in the long term, a recent paper advises researchers not to automatically ignore small effects due to their relevance of accumulation over time, and to be more sceptical about large effect sizes (Funder & Ozer, 2019). Small effects might have important consequences when repeated over time or, in the case of low self-esteem, when constantly maintained or unaccounted for. Therefore, further research is needed on whether self-esteem is a modifiable risk factor that can be targeted in behaviour change interventions, leading to a public health benefit.

A major strength of this study is the large sample size and nationwide recruitment. Studies with large sample sizes are more likely to produce accurate and less confusing results (Funder & Ozer, 2019). However, it also presents a few limitations. The generalisability of the findings may be limited as the sample was not found to be representative of the UK population (Rentfrow et al., 2015). Therefore, it is difficult to compare the findings of the current study with previous literature and the prevalence of smoking and excessive alcohol consumption reported in the UK due to the disproportionately high percentage of female participants (67.9%), individuals with high SES (61.6%) and those with low depressed mood (72.5%) within the sample. Such a large difference in certain sociodemographic characteristics may lead to potentially ambiguous results, even though roughly half of the current sample had low self-esteem (53.9%).

Another limitation of this study is that it cannot establish whether these associations are causal, and if so, what direction. This will require further research such as longitudinal studies to understand whether a causal relationship exists. Nonetheless, this study provides an early insight of the role self-esteem may play in smoking and excessive alcohol consumption.

The measures used represent another limitation of the study. A single-item measure was used to assess self-esteem and it was operationalised as a binary construct (i.e. low vs. high); therefore, the potential richness of the quality of people’s self-esteem might not have been captured (e.g. whether someone had a secure or fragile high self-esteem). Similar issues can be raised regarding the measures of the depressed mood, where the cut-off is arbitrary and information might be lost (Ranganathan, Pramesh, & Aggarwal, 2017). Furthermore, the BBC Lab UK database did not include well-established alcohol consumption measures, such as the AUDIT, therefore, the comparability of the results from this study with those in the relevant literature is limited. Furthermore, whilst this study adjusted for a number of covariates available in the dataset, there may have been other possible confounders that would have influenced the outcomes (Plard et al., 2015) that could not be adjusted for.

## 6 Conclusion

To our knowledge, this study was the first large study that assessed the associations between self-esteem and smoking and alcohol consumption among the adult population in the UK. The results showed a small association between lower self-esteem and smoking, and excessive alcohol consumption, and between higher self-esteem and current drinking. Future research is needed to better investigate the role self-esteem may play in these health behaviours and to examine whether there is a causal relationship between self-esteem and smoking and self-esteem and excessive alcohol consumption, and if so, what is their direction.

## Funding

This research did not receive any specific grant from funding agencies in the public, commercial, or non-profit sectors.

## Declaration of Competing Interest

Authors DS, IT, CG and NB do not have any conflicts of interest. RW has received travel funds and hospitality from and undertaken research and consultancy for, pharmaceutical companies that manufacture and/or research products aimed at helping smokers to stop.
